# A Non-Linear Deterministic Model for Regulation of Diauxic Lag on Cellobiose by the Pneumococcal Multidomain Transcriptional Regulator CelR

**DOI:** 10.1371/journal.pone.0047393

**Published:** 2012-10-22

**Authors:** Alessandro Boianelli, Alessandro Bidossi, Luciana Gualdi, Laura Mulas, Chiara Mocenni, Gianni Pozzi, Antonio Vicino, Marco R. Oggioni

**Affiliations:** 1 Center for Complex Systems Studies (CSC), Department of Information Engineering, University of Siena, Siena, Italy; 2 LAMMB, Department of Biotechnology, University of Siena, Siena, Italy; 3 UOC Batteriologia, Azienda Ospedaliera Universitaria Senese, Siena, Italy; University of Florida, United States of America

## Abstract

When grown on glucose and beta-glucosides, *S. pneumoniae* shows sequential use of sugars resulting in diauxic growth with variable time extent of the lag phase separating the biphasic growth curve. The pneumococcal beta-glucoside uptake locus containing the PTS transporter spr0276-82, is regulated by a multi-domain transcriptional regulator CelR. In this work, we address the contribution of phosphorylation of the phosphorylable cysteine in the EIIB domain of CelR to diauxic lag. Utilising site-directed mutagenesis of the phosphorylable amino acids in the EIIB and EIIA domains of CelR, we show that the EIIB domain activation is linked to the duration of the lag phase. Analysis of mutants for other PTS systems indicates that a second beta-glucoside PTS (spr0505), not able to support growth on cellobiose, is responsible for the lag during diauxic growth. A mathematical model of the process is devised together with a nonlinear identification procedure which provides model parameter estimates characterizing the single phases of bacterial growth. Parameter identification performed on data recorded in appropriate experiments on mutants allows for establishing a relationship between a specific model parameter, the EIIB domain and the time extent of the diauxic lag. The experimental results and the related insights provided by the mathematical model provide evidence that the conflicting activation of the CelR regulator is at the origin of the lag phase during sequential growth on glucose and cellobiose. This data is the first description of diauxic lag regulation involving two PTS and a multidomain regulator and could serve as a promising approach for studying the *S. pneumoniae* growth process on complex carbon sources as possibly encountered in the human host.

## Introduction

*Streptococcus pneumoniae* (pneumococcus) is a community acquired human respiratory pathogen responsible of important life-threatening invasive diseases such as pneumonia, meningitis, and bacteremia, as well as other less serious but very frequent infections, such as otitis media. More commonly, pneumococcus colonizes the nasopharynx mucosae asymptomatically, a process that occurs in the very first few months of life [1]. The pneumococcus is strictly fermentative, and sugars are the only sources of energy for biosynthesis and growth. Carbohydrates are therefore crucial for *in vivo* fitness governing a large number of processes, including virulence and progression to the disease [2]–[4]. This is well reflected by the large number of genes deputed to carbohydrate uptake systems and metabolic enzymes, accounting for a large fraction of the pneumococcal chromosome, which is often not part of the core genome. A functional genomic analysis of carbohydrate uptake in pneumococci was performed in [5], where we identified at least thirty two fermentable carbon sources at the occurrence of twenty-one phosphotransferase systems (PEP-PTS, phosphoenolpyruvate:sugar phosphotransferase system), seven carbohydrate uptake ABC transporters, one sodium:solute symporter and a permease.

**Figure 1:**
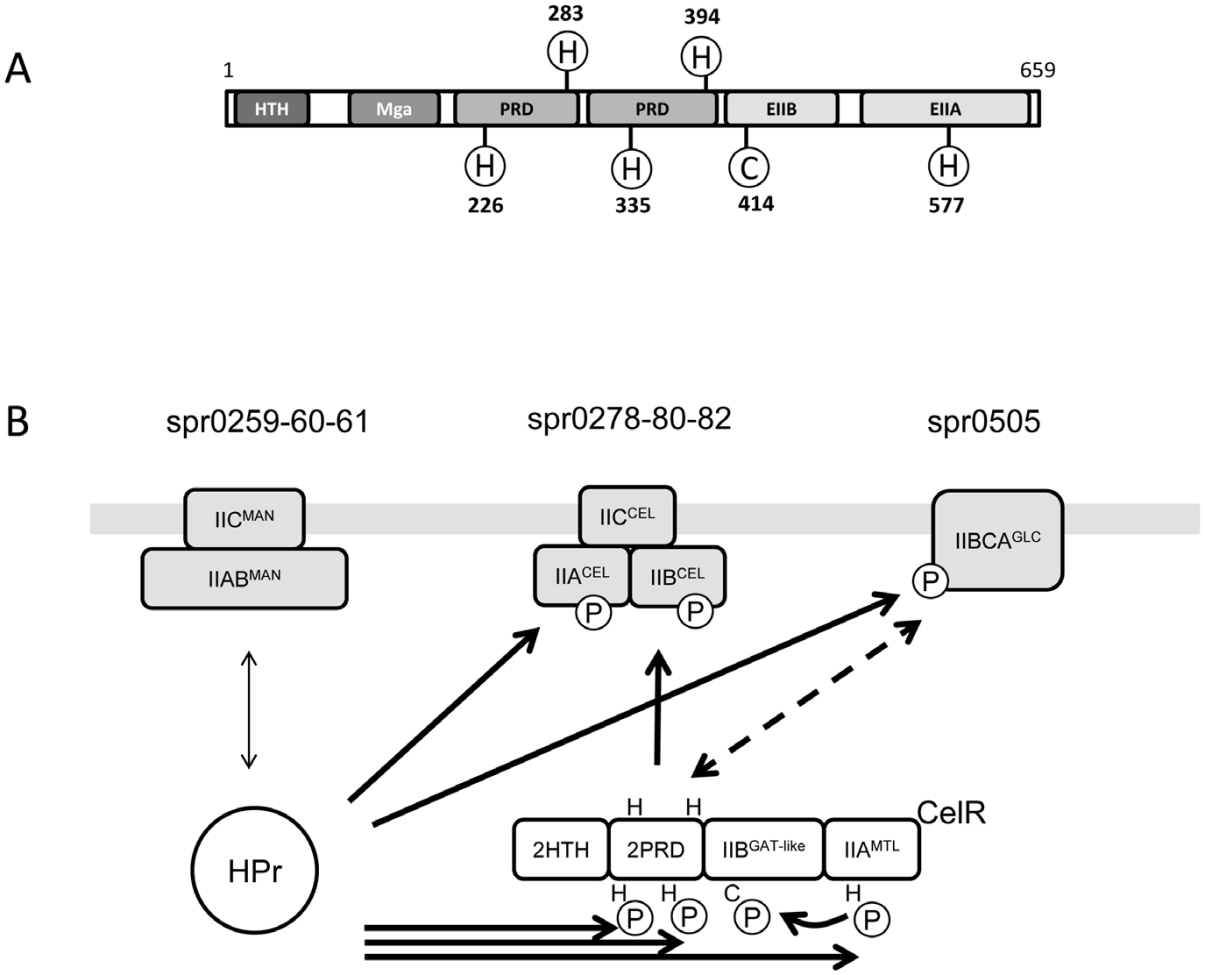
Schematic drawing of the CelR regulator and proposed model for regulation. Panel (A) shows the CelR regulator composed of a helix-turn-helix (HTH) domain, a M trans-acting positive regulator (Mga) domain, two PTS-regulatory domain (PRD), and phosphotransferase system EIIB^Gat-like^ and EIIA^Mtl^ domains [26]. The phosphorylable conserved residues, five histidine and one cysteine, are shown [8], [17]. Panel (B) shows the proposed regulatory circuit in presence of cellobiose and absence of glucose. In such a situation in *S. mutans* HPr protein does not phosphorylate the glucose transporter (pneumococcal orthologue spr0259-60-61), but phosphorylates the cellobiose PTS (pneumococcal orthologue spr0278-80-82) and the CelR regulator [8]. In addition the cellobiose PTS does also dephosphorylate CelR [8]. Phosphorylation in presence of cellobiose of the second beta-glucoside PTS [5] is in accordance with gene expression data (Safeeq and Kuipers, personal communication). Phosphorylation of the CelR EIIB domain phosphorylable cysteine by the CelR EIIA domain is deduced from our growth phenotypes. The putative interaction of the spr0505 EIIBA domains with the PRD domains of CelR is shown as dashed line.

Bacterial carbohydrate uptake operons are generally functional units and include, in addition to the transporter genes also genes for glycosyl-hydrolases for generation of mono- or disaccharides, enzymes for the metabolic steps linking the specific sugar to glycolysis and usually a regulator. One of the best characterized operons of *S. pneumoniae* is the spr0278-80-82 lactose type PTS (TC_4.A.3) for beta-glucosides [6], [7]. This PTS transporter is composed of three separate subunits: CelB (EIIB, spr0278, NP_357872), CelC (EIIA, spr0280, NP_357874), and CelD (EIIC, spr0282, NP_357876) within an operon containing also a multidomain transcriptional regulator and the BglA beta-glucosidase [6]–[8]. We recently characterized the substrate affinity of this transporter and it turned out to be responsible for the uptake of beta-glucosides cellobiose, gentiobiose, arbutin, amygdalin and aesculin [5]. Interestingly, growth on glucose and beta-glucosides showed sequential use of sugars resulting in diauxic growth [5], [9]. The diauxic growth phenomenon discovered by J. Monod is one of the most classical examples of the optimal nature of microbial regulatory processes [10]. When an organism is exposed to two substrates of carbon and energy source, it first consumes the substrate which supports the more efficient growth rate. Only after the more efficient growth-supporting substrate is virtually exhausted, bacteria start to synthesize the enzymes which belong to transport system necessary for the utilization of the second substrate [11]. In this situation, the diauxic growth curve presents three phases: the initial exponential phase, where the organism uses the preferred carbon source; a second phase named “diauxic lag”, where the synthesis of enzymes necessary for transport and utilization of the secondary carbon source is repressed [11]: in this phase the organism is not growing. The third phase is the exponential phase where, after the derepression of the operon for the alternative sugar, the organism utilizes the secondary carbon source. In the classical model for diauxic growth, increasing concentrations of the less preferred carbon source reduce the lag period between the two exponential growth phases [10], [12]–[14]. In contrast, increasing concentrations of beta-glucosides for *S. pneumoniae* cause an increase of the lag period during diauxic growth on glucose and beta-glucoside substrates [9]. Such behaviour appears to be an exception, being in contrast to the theory that links an increase of inducer to a decrease in lag time. One of the few examples is *Lactobacillus plantarum,* where increased lactose concentrations correlate with increased diauxic lag [15]. Previous work on the streptococcal beta-glucoside operon did focus on a detailed description of the multidomain regulator and regulation at the transcriptional level [7], [8], [16], not describing diauxic growth which occurs at low cell density [9]. The main player in the regulation of this locus in both the pneumococcus and *S. mutans* is the multidomain transcriptional regulator CelR (spr0279, NP_357873.1), which contains a N-terminal helix-turn-helix domain, followed by a MgA domain, two PRD domains (PTS Regulation Domain), a PTS EIIB domain and a PTS EIIA domain, both of the mannitol family (TC_4.A.2) [8] (Figure 1). Within these domains there are six amino acids that can be phosphorylated for regulation and which include H226 and H283 in PRD_1, H335 and H394 in PRD_2, C413 in PTS_EIIB and H577 in EIIA (Figure 1). The contribution of histidine phosphorylation in the two PRD and EIIA domains has been characterised in the *S. mutans* orthologue [8]. In the published model, in absence of glucose HPr activates the regulator by phosphorylating two histidines in the PRD domains and the one histidine in the EIIA domain, while in the presence of cellobiose the EIIA^Cel^ domains of the transporter activate the regulator by de-phosphorylating the other two histidines of the PRD domains [8]. A third regulatory mechanism by the EIIA^Man^ domain of the main glucose transporter has been found to repress the regulator in the presence of glucose [8] (Figure 1). The *S. mutans* CelR regulator is thus a case where, in addition to HPr, two different PTS systems have opposing regulatory influence on the same regulator [8]. Information on the importance of the EIIB domain of the regulator was so far only available from the MtlR activator of the mannitol operon in *Bacillus subtilis* [17]. In this EIIB domain, cysteine phosphorylation has been shown to have an important role in *mtl* operon induction [17].

In this work we address the contribution of phosphorylation of the phosphorylable cysteine in the EIIB domain of the CelR multidomain regulator and in particular its role in determining the time extent of the length of the diauxic lag. Analysis of mutants is performed by exploiting a nonlinear dynamic mathematical model for the diauxic growth and a nonlinear identification procedure providing parameter estimates characterizing the single phases of the bacterial growth. The model proposed in this work consists of the extension of our previous models [9]. The analysis of experimental data recorded in several experiments performed on mutants of CelR and different PTS systems allows for a dynamical interpretation of the process, provides additional evidence for the relevance of EIIB phosphorylation for *celB* and supports the hypothesis of a biological mechanism leading to the regulatory conflict that determines the lag in pneumococcal growth on beta-glucosides.

## Results

### Biphasic Growth on Beta-glucosides

**Figure 2:**
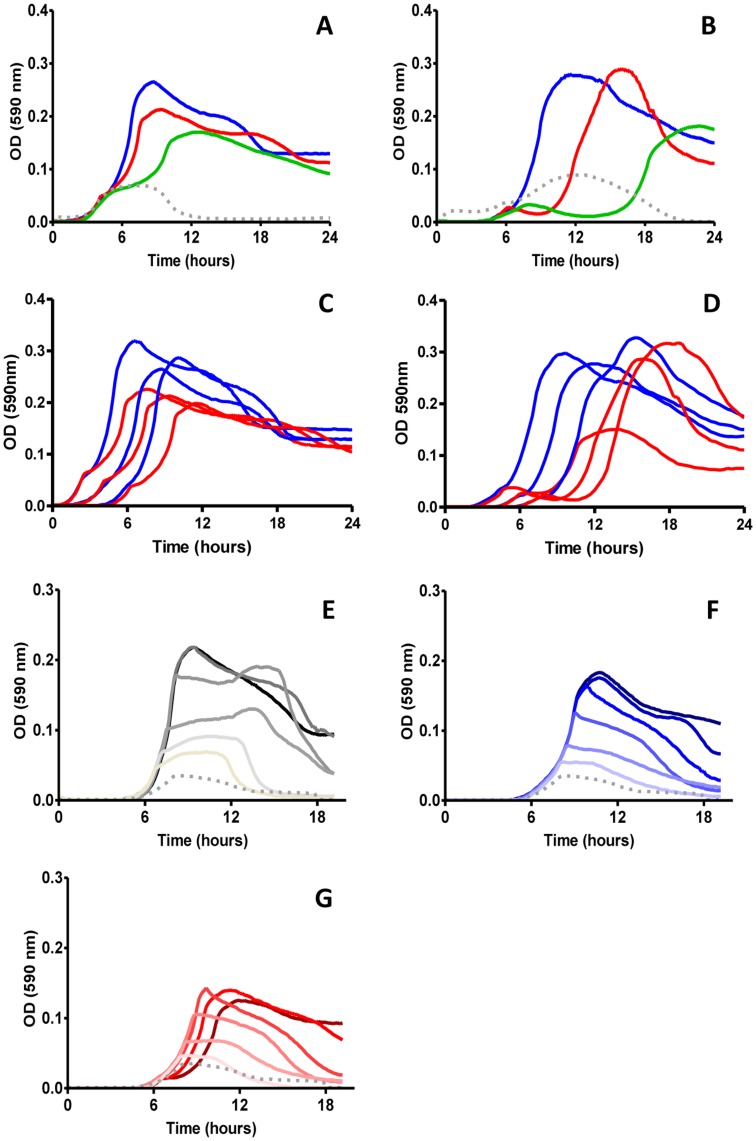
Pneumococcal growth in liquid medium containing different beta-glucosides. DP1004 (panel A) and a spr0278-80-82 positive G54 derivative (panel B) were recovered from agar plates, resuspended to OD_660_ = 0.2 and inoculated in CAT medium containing one of the following beta-glucosides as sole added carbohydrate (1% w/v): gentiobiose (blue), cellobiose (red) and amygdaline (green). Panel C and D report on growth curve performed on serial dilutions of starting inoculum of strain DP1004 (C) and the spr0278-80-82 positive G54 derivative (D). Panels E to G show pneumococcal strain DP1004 grown in CAT medium containing two fold serial dilutions of sugars ranging from 0.5% w/v to 0.015% (decreasing colour intensity): glucose (panel E; black), gentiobiose (panel F; blue) and cellobiose (panel G; red). The grey dashed lines represent growth of bacteria in CAT medium without sugar.

*S. pneumoniae* is able to utilize a variety of beta-glucosides as sole carbon source for growth, most of which are imported and metabolized via the spr0274-spr0282 operon [5], [6]. When grown in a peptone yeast extract medium (CAT medium) with added gentiobiose, the rough D39 derivative DP1004 grew readily with a generation time similar to that in media containing glucose. In contrast, cells grown on cellobiose or amygdalin showed biphasic growth indicative of sequential use of carbon sources (Figure 2A), due to the residual presence of glucose in the CAT medium, as indicated in the Materials and Methods paragraph. In cellobiose, the lag time was one hour, after which the strain grew with a similar doubling time as in gentiobiose-medium. The lag phase in amygdalin containing medium was even longer than that for cellobiose, with a growth arrest of up to four hours. When increasing the concentration of glucose and gentiobiose as sole carbon source in the medium, the only measurable effect was the increase in maximal cell density. Differently increasing concentrations of cellobiose (Figure 2F) and amygdalin (not shown) lead also to a proportional increase in the extension of the lag period.

To rule out that the repression after the first growth phase was dependent on residual sugar in inoculum, we performed serial dilutions of inocula. With the exception of proportional delays of growth initiation, this data showed unaltered biphasic growth profiles (Figure 2C). Growth on serial dilutions of glucose and fixed concentrations of cellobiose showed that the cellular density reached during the initial growth phase was clearly dependent on the amount of glucose and that glucose concentrations of 0.015% mimicked growth behaviour on unsupplemented CAT (data not shown). These data indicate that initial growth depends on the nutrient present in unsupplemented CAT and that it is independent from possible sugars present in the inoculum.

Marker-less transfer of the spr0274-spr0282 operon into G54, a serotype 19F strain devoid of this operon, allowed growth of the recombinant in gentiobiose, cellobiose and amygdalin. Albeit some growth parameters were different between the original host of the operon and the recombinant, the most striking feature regarding biphasic growth on beta-glucosides was maintained (Figure 2B).

### Role of the EIIA and EIIB Domains in Activity of the Beta-glucoside Regulator

**Figure 3:**
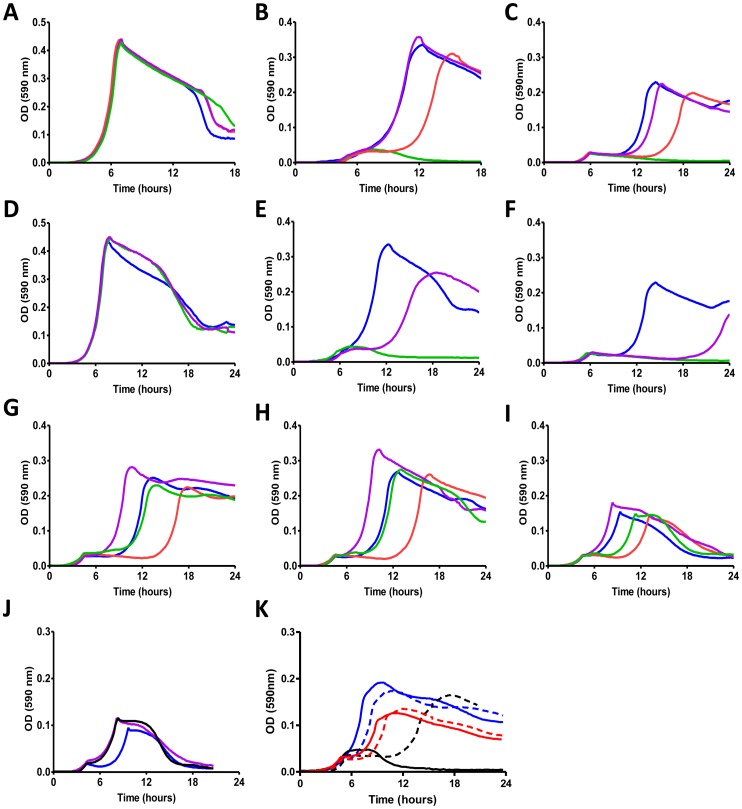
Growth of mutants in the EIIB^Man^ and EIIA^Man^ domains of the CelR beta-glucoside regulator. Wild type strain (blue) and a its isogenic mutants in the regulator EIIB domain (panels A, B and C) and EIIA domain (panels D, E and F) were collected from agar plates, resuspended to OD_590_ of 0.2 and inoculated in CAT medium containing one of the following carbohydrates: glucose 0.3% (panels A and D), gentiobiose 0.3% (panels B and E) and cellobiose 0.3% (panels C and F). Pneumococcal strains were DP1004 (blue, all panels), EIIB domain knockout (green, panel A, B and C), EIIB_C414A_ mutant (red, panel A, B and C), EIIB_C414D_ mutant (violet panel A, B and C), EIIA domain knockout (green, panels D, E and F) and EIIA_H577A_ mutant (violet, in panels D, E and F). Panel G through I report growth in 1%, 0.3% and 0.1% cellobiose respectively of the *wt* (blue), the EIIB_C414A_ mutant (red as in panels A-D), the EIIBCA^Glc^ spr0505 mutant (violet), and the EIIB_C414A_ and EIIBCA^Glc^ spr0505 double mutant (green). Panel J shows the wt (blue), the EIIBCA^Glc^ spr0505 insertion mutant (violet as in panels G-I) and the spr0505_EIIA in frame deletion mutant (black) grown in 0.1% cellobiose. Panel K shows growth of our wt strain in gentibiose (continuous blue), gentibiose plus methyl-beta-glucoside (dashed blue), cellobiose (red), cellobiose plus methyl-beta-glucoside (dashed red), methyl-beta-glucoside only (black dashed), and without sugar (black). All sugar concentrations in panel K are 0.2%.

A recent study demonstrated, in addition to the phosphorylation state of the PRD and EIIA domains, also involvement of the EIIB domain of the *B. subtilis* MtlR regulator in mannitol operon induction [17]. The presence of an identical domain in spr0279, brought us to investigate the role of this EIIB domain in the pneumococcal multi-domain regulator CelR. In order to determine its involvement in beta-glucoside metabolism, we generated point mutations in the predicted phosphorylation site of the EIIB (Table 1). Three isogenic mutants respectively with a substitution of the phosphorylable cysteine by an alanine (predicted to mimic an unphosphorylated residue), a substitution of the phosphorylable cysteine with an aspartic acid (predicted to possibly mimic a phosphorylated residue) and a mutant with a CelR lacking EIIB, were tested for their capacity to grow in CAT medium supplied with either glucose (0.3%), gentiobiose (0.3%) or cellobiose (0.3%). None of the mutants differed from the *wt* in their capacity to grow in glucose containing medium (Figure 3A). The truncation of the regulator (deletion of the entire EIIB domain) resulted in a complete incapacity to grow both in gentiobiose and cellobiose (green line in Figure 3BC). The change of Cys414 with an alanine residue allowed FP411 still to metabolise beta-glucosides, but the growth curve showed a significant increase of the lag phase. This phenotype was more pronounced in cellobiose than in gentiobiose (red line in Figure 3BC). In contrast, the replacement of Cys414 with an aspartic acid residue, resulted in a wild type behaviour (violet line Figure 3BC).

**Table 1:** Strains and isogenic mutants in the EIIB and EIIA domains used in this study.

Strain	Genotype	Description	References
DP1004	*rpsL41*	Sm^R^	[20], [21]
FP410	*rpsL41,* spr0279::*aphIII*-*rpsL^+^*	EIIB ko, Km^R^ and Sm^S^	This work
FP411	*rpsL41,* spr0279_TGT1240-2GCC_	EIIB_C414A_, Sm^R^	This work
FP439	*rpsL41,* spr0179_TGT1240-2GAC_	EIIB_C414D_, Sm^R^	This work
FP415	*rpsL41,* spr0279::*aphIII*-*rpsL^+^*	EIIA ko, Km^R^ and Sm^S^	This work
FP440	*rpsL41,* spr0279_CA1729-30GC_	EIIA_H577A_, Sm^R^	This work
FP376	*rpsL41,* spr0505::*aphIII*	PTS^Glc^ spr0505 ko, Km^R^ and Sm^R^	[5]
FP302	*rpsL41,* spr1834::*aphIII*	PTS^Lac^ Spr1834 ko, Km^R^ and Sm^R^	[5]
FP463	*rpsL41,* spr0505::*aphIII*, spr0279_TGT1240-2GCC_	PTS^Glc^ Spr0505 ko and EIIB_C414A_, Km^R^ and Sm^R^	This work

In order to comprehend the involvement of the PTS-EIIA domain spr0279 function, we focused our interest on its phosphorylable His577 [17], [18]. As described above, we constructed first an EIIA deletion mutant (FP415) and then a recombinant with a His-Ala replacement in position 577 (Table 1). As for the spr0279 EIIB truncation also the EIIA truncated mutant did not grow on beta-glucosides. The replacement of His577 with an alanine in EIIA resulted in an interesting phenotypic effect combining the increase in the diauxic lag observed for the EIIB Cys414Ala mutant with a decrease in generation time when grown on beta-glucoside substrates (violet line in Figure 3EF).

Since more than one PTS has been shown to influence CelR in *S. mutans,* we analysed the growth profile of mutants for the main glucose PTS spr0259-60-61, and mutants for the two other predicted beta-glucosides PTS transporters spr0505 and spr1834-5-6 [5]. No changes in growth profiles on cellobiose were observed for mutants of the main glucose/mannose transporter spr0259-60-61 and the third putative beta-glucoside transporter spr1834-5-6 (data not shown). On the contrary the mutant for the spr0505 PTS (FP376) grew on cellobiose with nearly no lag period between initial growth and growth on cellobiose and this short lag was cellobiose concentration independent (Figure 3G–I). As in the case of the *wt*, transformation of the CelR_EIIB-Cys414Ala mutation into the spr0505 mutant led to increase of lag with increasing duration at increasing cellobiose concentrations (Figure 3G–I). The insertion mutant which deletes the whole spr0505 disrupts also the transcriptional unit with the downstream beta-glucosidase. As a control we have constructed a in frame mutant for only the EIIA domain of spr0505. The identical growth behaviour in cellobiose of the two mutants indicates the specificity of the phenotype observed (Figure 3J). In previous work we associated 1-O-Methyl-beta-glucoside uptake to transporter spr0505 (SP0577 in TIGR4) [5]. Growth on this sugar alone shows a very long lag time and here we show that addition of methyl-beta-glucoside to medium containing either gentibiose or cellobiose generates or extends respectively the lag time during growth also on these sugars. This is in accordance with the proposed action of the spr0505 on CelR.

### Mathematical Model Formulation and Parameter Identification

A non-linear deterministic model, describing pneumococcal diauxic growth on beta-glucosides, is proposed for the characterization of diauxic lag. The model is grounded on previous model developed for diauxic growth on beta-glucosides [9], which has been suitably extended by introducing a new hill function taking into account the inhibition effect of beta-glucosides on transcriptional induction of PTS enzymes.

**Figure 4:**
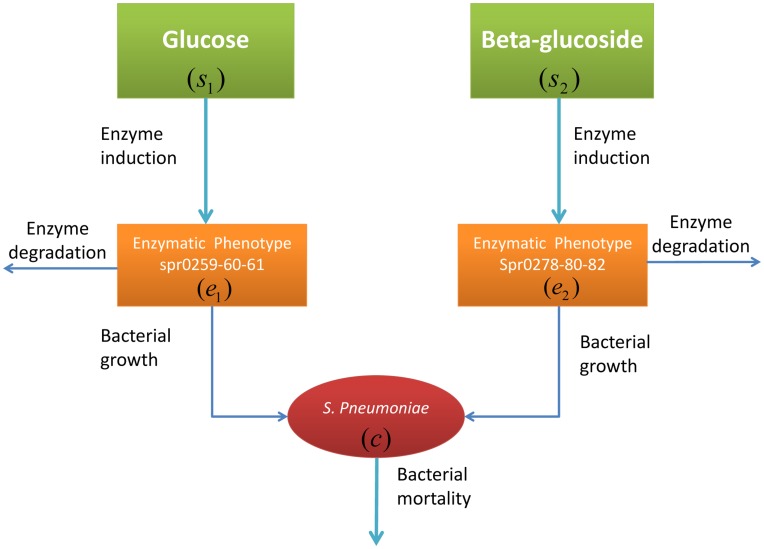
Schematic representation of the dynamic model. The glucose and beta-glucoside substrates are supplemented initially at a certain concentration. The enzymes spr0259-60-61 and spr0278-80-82 are induced by glucose and beta-glucoside substrates respectively through transcriptional regulation. The *S. pneumoniae* growth process is sustained by glucose and beta-glucoside through enzymes spr0259-60-61 and spr0278-80-82.

Figure 4 illustrates the chemical species and the main reactions involved in the model. Specifically, we consider the following model variables:

- Bacteria: we denote by *c* the *S. pneumoniae* concentration (mass per unit volume of culture) present in the media;
- Enzymes: the enzymes spr0259-60-61 for glucose and spr0278-80-82 for beta-glucoside, both belong to PEP-PTS system. They are treated separately from the bacteria variable, since they serve as catalysts for reactions between bacteria and substrates [11]. We denote the enzyme concentrations for glucose and beta-glucoside transport, normalized with respect to gram dry weight (gdw), by
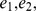
respectively;
- Substrates: we consider the substrate concentrations of glucose and beta-glucoside as extracellular components. We denote them by 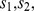 respectively.

The glucose and beta-glucoside substrates are introduced initially in the media with fixed concentration. Their uptake by the *S. pneumoniae* is assumed to be catalyzed by the set of PTS system enzymes (spr0259-60-61 for glucose and spr0278-80-82 for beta-glucoside, respectively).

**Figure 5:**
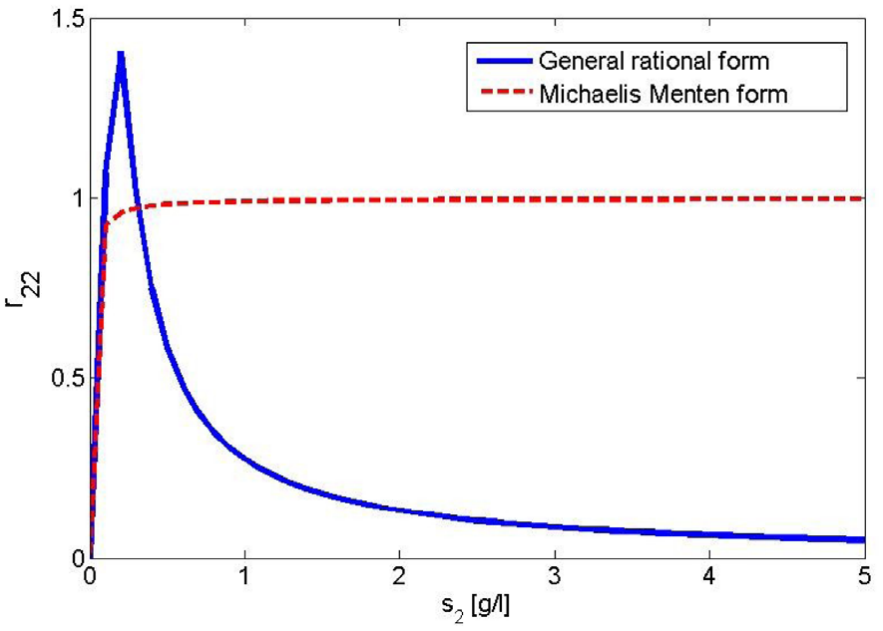
Graphical representation of the general rational form (blue) and Michaelis - Menten form of function 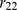 (red line). We represent here the main difference between these two functions. Note that at high concentrations of beta-glucoside the growth rate is negligible, while it grows when the beta-glucoside concentration 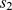 decreases, becoming similar to the Michaelis Menten form adopted in the previous diauxic growth model.

The transcription of the enzymes spr0259-60-61 and spr0278-80-82 is activated by the presence of glucose and beta-glucoside. Model equations describing the dynamic evolution of enzyme, bacteria and substrate concentrations together with related reaction rates have been derived on the basis of mass balance and mass action laws. The basic structure of the model equations is as follows:
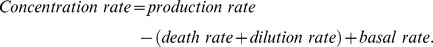



This formalism leads to a system of nonlinear ordinary differential equations (ODE). More specifically, we introduce the growth rates for enzyme induction :
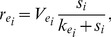
(1)where 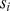 is the concentration of substrate *i*, 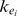 is the Michaelis-Menten constant for the enzyme 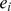 and 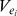 is the maximum rate for enzyme induction. Moreover, the growth rates of bacteria are as follows:
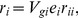
(2)where, for the first substrate 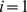:
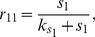
(3)and for the first substrate 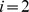:



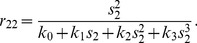
(4)In equation (2), 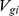 is the maximum growth rate for bacterial production *c* on the substrate 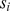, and 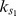
in equation (3) is the Michaelis-Menten constant of the first substrate. The coefficients 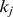, 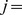
0, 1, 2, 3 in equation (4) are parameters of the inhibition hill function. Actually, the choice of the functional form for 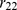 follows from experimental observations, indicating that the diauxic lag increases with increasing initial betaglucoside concentration; the bacterial growth rate in the third phase of the process (“slowdown phase”) decreases smoothly with the cellobiose concentration. Figure 5 shows the function 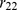 compared to a traditional Michaelis-Menten form.

Since multiple substrates are present, the cellular regulatory processes of inhibition/activation and repression/induction affect rate equations. The effect of this regulation is represented by the control variables 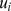 and 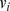. The variables 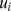 represent the fractional allocation of resource for the synthesis of enzymes 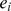. Through these variables, the model predicts control on inhibition or activation of PTS enzyme transcription for transport and utilization of substrate 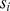. According to the matching law, 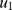 is defined as [19]:
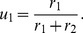
(5)


**Figure 6:**
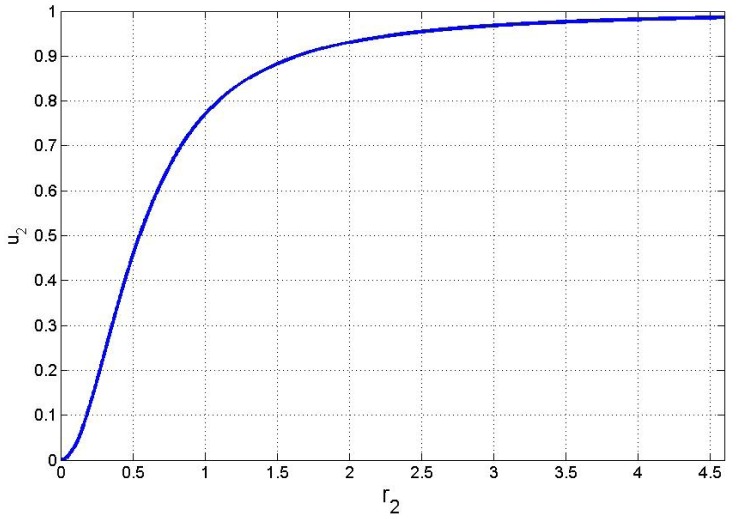
Graphical representation of the Hill function form (blue) for 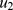 control variable. For lower values of 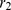, the variable presents values nearly to zero. Otherwise for higher 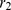 values, it reaches its asymptotic value 1.

The control variable 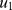 regulates the catabolite repression effect by the glucose presence on cellobiose PTS pathway. The control variable 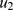
regulates the rate of PTS beta-glucoside enzyme synthesis. We introduce a hill function capturing the transcriptional inhibition of PEP PTS system enzyme spr0278-80-82 caused by the presence of the beta-glucoside. This assumption takes into account our main hypothesis regarding the lack of activation of spr0278 locus by CelR multidomain regulator (see Figure 1). The function 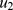
is defined as follows:
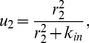
(6)where 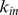 is the hill function constant and 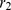 is the growth rate for the beta-glucoside defined in (2). Figure 6 shows a graphical representation of the function 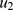 in equation (6). Therefore, the growth rates for the enzyme synthesis can be expressed as 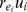.

The control variables 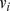 represent the regulation mechanism of the activity of enzymes 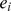, which in turn determines the bacterial growth rate. The mathematical definition of these variables, is [13]:
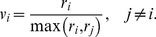
(7)


By taking into account the control effect of enzyme activity, the growth rates introduced in (2) become 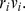


Since enzymatic efficiency of import and metabolic utilization is different in different substrates, we introduce the actual bacterial growth rate over the two substrates as:
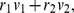
where 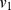 and 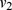 allow to model the switching mechanism between the two bacterial PEP-PTS in the sequential growth process on substrates 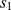 and 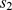. Indeed, it should be noted that the control variables 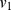 and 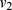 are dimensionless quantities, leaving invariant the growth rate meaning. By incorporating the effect of dilution of the specific enzyme level due to cell growth, constant enzymes decay in the cells and bacterial mortality, the state equation model can be written as:
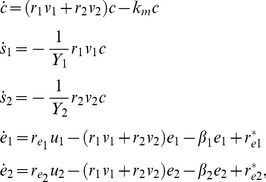
(8)where 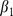, and 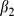 are first-order enzyme decay parameters, 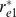 and 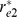 are the basal rates of enzyme synthesis, 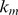 is the constant rate of bacterial mortality and 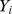 is the yield coefficient of Monod for the i-th substrate. The model parameters are listed in Table 2.

**Table 2:** Parameters of the model and their biological meaning.

Parameter	Biological Meaning	Units(SI)
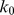	Polynomial coefficient	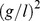
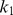	Polynomial coefficient	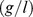
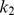	Polynomial coefficient	*adim*
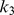	Polynomial coefficient	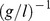
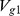	Maximum growth rate constant on glucose	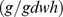
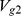	Maximum rate constant on beta-glucoside	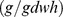
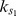	Michaelis Menten constant for glucose	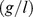
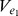	Maximum rate of enzyme production for glucose	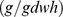
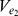	Maximum rate of enzyme production for beta-glucoside	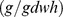
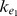	Michaelis Menten constant for enzymes of glucose	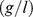
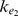	Michaelis Menten constant for enzymes of beta-glucoside	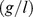
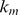	Bacterial mortality rate	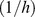
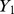	Yield coefficient of Monod for glucose	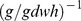
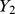	Yield coefficient of Monod for beta-glucoside	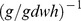
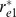	Constitutive rate of enzymes synthesis for glucose	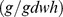
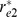	Constitutive rate of enzymes synthesis for beta-glucoside	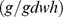
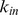	Hill constant	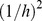
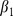	Enzyme decay constant for glucose	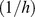
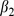	Enzyme decay constant for beta-glucoside	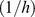

The mathematical model (8) has been identified by estimating its parameters on the basis of the experimental data described in the previous sections. The values of the estimated parameters, for *wt* and mutants both on the cellobiose and gentibiose concentrations of 3 g/l, are reported in Tables 3, 4. Estimated parameters for *wt* DP1004 with serial dilutions of both cellobiose and gentiobiose are reported in Tables 5, 6.

**Table 3:** Parameters estimation with cellobiose concentration 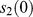 = 3 g/l.

Parameter	DP1004	FP411		FP439	FP440	FP376	FP463
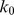	0.109		0.107	0.0734	0.135	0.122	0.141
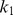	0.391		0.372	0.343	0.368	0.325	0.347
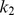	−1.09		−1.15	−1.39	−1.05	−1.31	−1.67
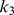	12.6		32.9	22.6	36	10.7	11.6
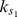	0.00146		0.0139	0.00121	0.00175	0.00131	0.00250
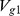	2833		2610	3072	2650	2576	2780
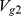	2846		2154	2895	1936	2500	2530
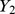	0.62		0.56	0.52	0.63	0.78	0.64
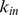	0.0675		0.164	0.153	0.100	0.159	0.113

**Table 4:** Parameters estimation with gentiobiose concentration 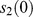 = 3 g/l.

Parameter	DP1004	FP411	FP439	FP440
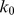	0.0045	0.134	0.130	0.113
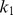	0.502	0.518	0.166	0.286
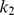	−1.44	−1.08	−1.90	−0.928
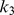	2.61	4.38	2.64	5.17
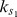	0.00209	0.00176	0.0006	0.000985
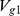	2000	1993	1927	1800
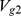	3151	2883	2801	2110
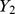	0.89	0.77	0.94	0.65
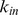	0.310	0.119	0.710	0.4

**Table 5:** Parameters estimation of strain DP1004 with dilutions of cellobiose concentration 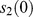 = 10 g/l, 3 g/l, 1 g/l and 0,3 g/l.

Parameter	10 g/l	3 g/l	1 g/l	0,3 g/l
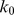	0.377	0.194	0.2359	0.0267
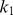	0.404	0.760	0.663	0.557
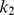	−2.77	−0.411	−1.67	−1.34
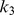	10.78	3.67	2.54	1.77
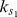	0.00176	0.00176	0.000582	0.000209
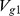	3630	3606	3338	2483
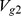	1851	1972	1876	2110
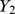	0.154	0.64	0.65	0.588
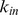	0.2875	0.0508	0.400	0.390

**Table 6:** Parameters estimation of strain DP1004 with dilutions of gentiobiose concentration 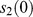 = 10 g/l, 3 g/l, 1 g/l and 0,3 g/l.

Parameter	10 g/l	3 g/l	1 g/l	0,3 g/l
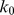	0.439	0.1702	0.273	0.214
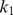	0.384	1.07	0.728	0.327
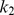	−1.33	−1.64	−2.89	−1.03
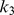	0.314	0.148	0.041	0.005
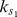	0.00648	0.00275	0.00101	0.00363
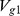	3066	3014	3599	3229
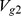	2060	1893	2053	2386
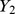	0.20	0.27	0.57	0.45
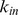	0.210	0.146	0.028	0.1

**Figure 7:**
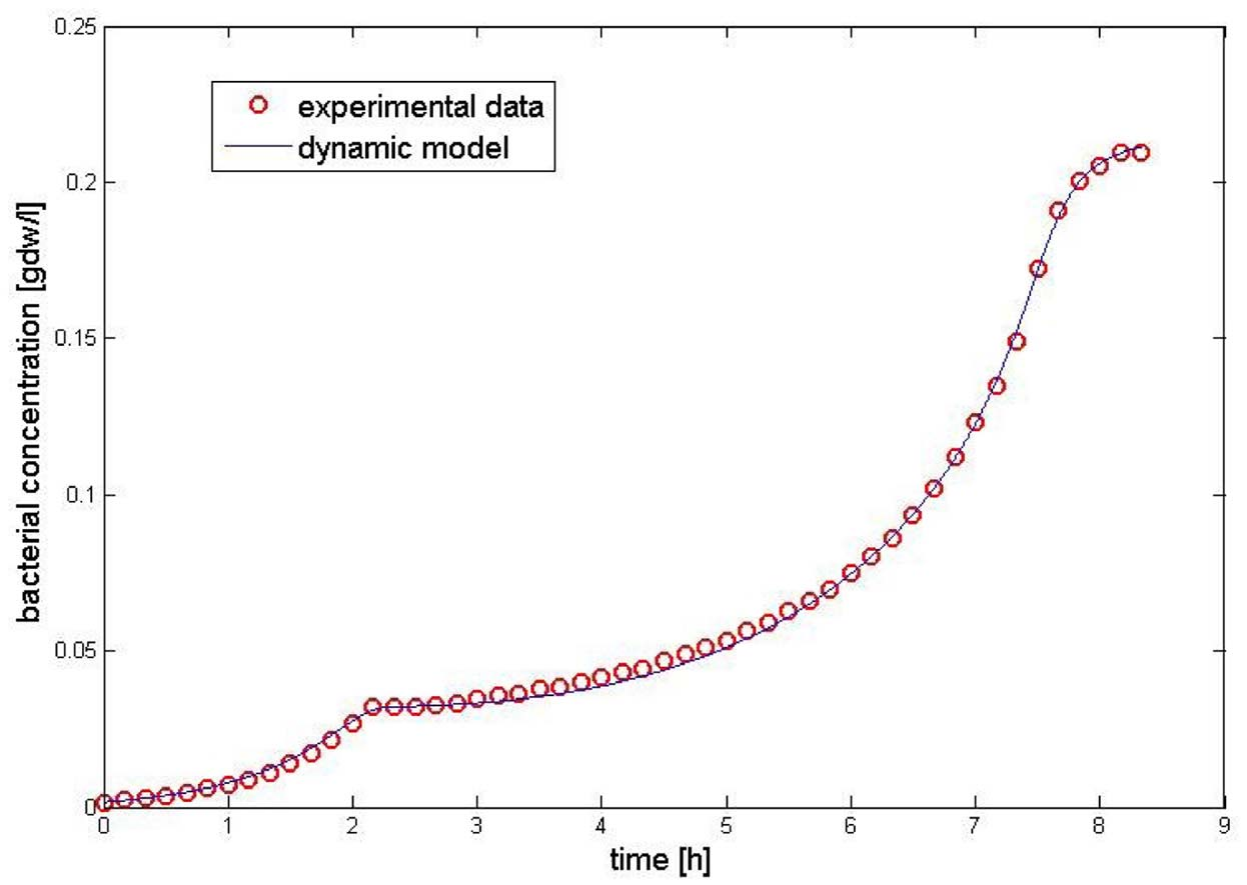
Fitting results of the dynamic model. Experimental data (red circles) and simulations provided by the fitted model (blue line) for wild type at initial concentration of 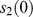 = 0.3 g/l. The fitting results look very good supported by the low value of cost function 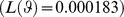.

Finally, the values of estimated parameters for FP411, FP376 and FP463 mutants with dilution of cellobiose are reported in Tables 7, 8, and 9. A snapshot of experimental data and bacterial concentration predicted by the identified model is reported in Figure 7. In this case, the fitting quality looks quite satisfactory. Focusing on numerical values of parameters 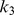 and 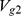, characterizing the diauxic lag duration and the maximum bacterial growth rate respectively, several comments are in order. First of all, with reference to *wt* DP1004, parameter 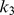, showed higher values on cellobiose than gentibiose for all serial dilutions considered in the experiments. Also, 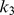 values decreased with decreasing initial concentration of cellobiose and gentiobiose. With reference to parameter 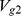, the *wt* didn’t show significant changes for all different initial concentrations. FP411 mutant showed higher values of the parameter 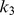 with respect to *wt* and mutants Fp376, FP463, for all serial dilutions. Moreover, FP411, FP376 and FP463 showed parameter 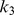 decreasing values for decreasing cellobiose initial concentrations. Regarding parameter 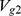, mutants FP411, FP376 and FP463 showed similar values for all concentrations used in the experiments. Finally, two experiments were performed setting cellobiose and gentiobiose initial concentrations at 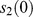 = 3 g/l. The results of the estimation procedure on FP440 data showed values of parameter 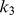 higher than other mutants and *wt.* Moreover, lower values of the parameter 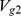 have been obtained with respect to other mutants and *wt*. Otherwise, FP376 showed lower values of the parameter 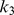 with respect to other mutants and *wt*.

**Table 7:** Parameters estimation of mutant FP411 (EIIB_C414A_) with dilutions of cellobiose concentration 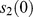 = 10 g/l, 3 g/l, and 1 g/l.

Parameter	10 g/l	3 g/l	1 g/l
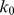	0.00432	0.004	0.0027
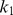	1.45	0.75	4.01
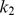	−2.23	−2.1	−1.40
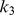	15.9	11.6	8.23
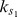	0.005	0.0134	0.00438
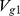	2952	3159	2740
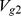	2806	2633	2851
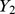	0.135	0.532	0.82
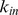	0.009	0.034	0.023

**Table 8:** Parameters estimation of strain FP376 (spr0505) with dilutions of cellobiose concentration 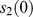 = 10 g/l, 3 g/l, and 1 g/l.

Parameter	10 g/l	3 g/l	1 g/l
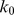	0.00407	0.00384	0.00135
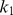	1.41	2.13	4.58
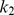	−2.36	−0.354	−1.43
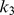	1.9	1.8	1.5
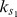	0.00133	0.00355	0.02
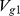	3220	3173	2753
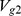	2800	2831	2987
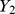	0.178	0.72	0.92
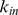	0.006	0.0547	0.0372

**Table 9:** Parameters estimation of strain FP463 (EIIB_C414A_ spr0505 double mutant) with dilutions of cellobiose concentration 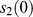 = 10 g/l, 3 g/l, and 1 g/l.

Parameter	10 g/l	3 g/l	1 g/l
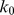	0.0075	0.0047	0.00272
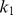	1.57	2.10	4.11
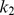	−1.28	−1.30	−1.52
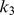	11.51	7.12	6.88
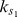	0.0047	0.004	0.0033
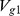	2990	3208	2719
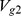	2608	2726	2870
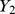	0.13	0.58	0.821
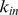	0.0144	0.130	0.05

## Discussion

Pneumococci are obligate fermentative organisms which utilise exclusively carbohydrates as source of carbon and energy [5]. A significant part of the pneumococcal genome is dedicated to carbohydrate metabolism and uptake, for which up to thirty different transporters have been recently described [5]. To allow for rational utilisation of sugars from the environment, the diverse carbohydrate uptake systems are tightly regulated in order to allow for sequential utilisation of carbohydrates, as already described in the model organisms *B. subtilis* and *E. coli* seventy years ago [10]. Growth profiles of pneumococci on different carbohydrates differ mainly for the doubling time of bacteria during exponential growth which ranges from 32 minutes on glucose to 640 minutes on ascorbate [5]. When monitoring growth on the beta-glucoside cellobiose in media containing also trace amounts of yeast derived carbohydrates, we observed a peculiar growth behaviour with a first rapid phase of growth followed after a short lag period by a second slower exponential growth [9]. Such growth pattern is termed diauxie [10], [11]. Our data here confirm, also by marker-less transfer of the whole spr0278-82 locus into another strain, that this growth behaviour is due to import of cellobiose by the well described beta-glucoside locus which is organised in two transcriptional units, one encoding a beta-glucosidase and a second one encoding a multidomain regulator and the subunits of a PTS transporter [6], [7]. In both strains the duration of the lag was dependent on the type of sugar, shortest or absent for gentibiose (glucose β1–6 glucose), short for cellobiose (glucose β1–4 glucose), and longer for amygdalin (gentibiose with a cyanide group), but most importantly, the extent of the lag phase was found to depend on sugar concentrations, with longer lag at higher beta-glucoside concentration. This is in contrast to the classical observations in *Bacillus subtilis* with xylose, *Klebsiella oxytoca* with arabinose or *Escherichia coli* with lactose, where sugar concentrations were inversely correlated to the duration of the lag phase [10], [12]–[14], which is mainly due to the obvious observation that the more inducer is present the faster the repression on the metabolic operon is relieved.

The main player in the regulation of the beta-glucoside operon is the multidomain regulator spr0279, which has been carefully described in the *S. mutans* CelR orthologue [8] (Figure 1A). Five histidines, located in the two PRD domains and in the EIIA domain, had been analysed in the *S. mutans* CelR orthologue by site directed mutagenesis aimed to abolish phosphorylation. This data showed that phosphorylation of the histidines in the PRD domains, corresponding to H283 and H394 of *S. pneumoniae* CelR, depends on the EIIAB^Cel^ domains of the spr0278-80-82 cellobiose transporter, while that of histidines H226, H335 in PRD and H577 in the EIIA domain depends on the global PTS activator HPr [8]. In order to probe the impact of this regulator in our growth phenotype, we constructed similar mutants, focusing our attention on the phosphorylable cysteine of the EIIB^Gat-like^ domain of the regulator, which had not been characterised in *S. mutans*, but recently found to be important in the MtlR regulator of *B. subtilis* [17]. Growth phenotypes conferred to recombinant strains were also analysed utilising a mathematical model developed to describe diauxic growth on beta-glucosides [9]. The unique opportunity conferred by the utilisation of this mathematical model is that it is possible to ascribe single parameters of the equation to specific biological phenomena and to define clearly which parameter is influenced and how significantly. The experimental data here show clearly that alanine substitution of the phosphorylable cysteine (C414) of the EIIB^Gat-like^ domain of the regulator (FP411 mutant) influences exclusively parameter 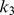 of our model, without impacting on any of the other parameters of the model. This aspect shows the crucial role of this parameter in the model. In fact, as pointed out in the results section the parameter 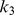 shows higher values for FP411 mutant with respect to DP1004 and this values decrease with decreasing cellobiose concentration [9]. This trends produce two crucial effects: the 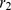 function values at the same cellobiose initial concentration is lower for FP411 mutant than *wt* indicating a better bacterial growth of the *wt*. This effect is less evident when the cellobiose concentration decreases. The second effect is relevant on the transcriptional regulation control variable 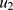 which shows lower values for high values of 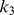. This aspect means that the transcriptional regulation of cellobiose enzymes in FP411 is repressed producing longer lag duration with respect to *wt*. We recall that this repression is less effective at lower cellobiose concentrations. As 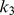 is the parameter correlated with the duration of the lag period, our data indicate that phosphorylation of the cysteine of the EIIB^Gat-like^ domain of the regulator impacts exclusively on lag duration, without influencing any other aspect of the regulation of the locus. Otherwise, the fact that alanine substitution of H577 of the EIIA^Mtl^ domain impacted both on parameter 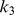 and 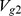 and thus on both lag duration and growth efficiency (maximum rate production constant) on beta-glucosides, indicates the probable direction of phosphor-transfer from the EIIA^Mtl^ to the EIIB^Gat-like^ domain of CelR (Figure 1B). The finding that the truncation of CelR after its PRD domains renders the operon silent is in keeping with a effect on the overall functionality of the regulator and explains also why in TIGR4, who shows a SNP in CelR separating the EIIA domain from the regulator, no cellobiose metabolism can be detected [5].

Having shown that EIIB^Gat-like^ is the key domain of CelR involved in duration of the lag period, we still lacked any indication on the regulatory conflict at the basis of this halt in regulation leading cellobiose-concentration dependent blockage of growth. Following indication from *S. mutans*, where glucose is reported to both induce CelR through the EII^Cel^ of its beta-glucoside transporter and at the same time to repress CelR via the EII^Man^ of the main glucose transporter [8], we evaluated impact of deletions of other pneumococcal PTS systems on cellobiose mediated growth lag. In contrast to *S. mutans*, deletion of the main glucose PTS spr0259-60-61, had no effect on growth on cellobiose [8]. Somewhat surprisingly the insertion mutant for the glucose type PTS spr0505 (FP376) and the EIIA in frame mutant of spr0505 showed a strongly reduced diauxic lag when grown on cellobiose, distinguishing the pneumococcal regulatory network from that of *S. mutans*. The pneumococcal PTS IIBCA^Glc^ spr0505 is cotranscribed with a beta-glucosidase and found to be linked to growth on methyl-beta-glucoside [5]. Albeit spr0505 does not allow growth on cellobiose in an spr0278-80-82 mutant [5], [6], its relationship to beta-glucoside metabolism is further documented by induction of transcription during the first minutes after termination of growth on glucose in presence of cellobiose (Safeeq and Kuipers personal communication). Differently from spr0278-80-82, the spr0505 PTS had been shown also to be subject to carbon catabolite repression [16]. The fact that the concentration dependent variation of the lag was abolished in the spr0505 mutants is in agreement with its possible role as beta-glucoside PTS with low affinity for cellobiose. Taken together these data indicate that immediately after termination of growth on glucose, the de-repressed and cellobiose-induced core-genome spr0505 PTS inhibits cellobiose uptake by spr0278-80-82 in a cellobiose dependent manner (Figure 1B). Our data do not resolve how the CelR EIIB domain and spr0505 EIIBA domains extend the lag during diauxic growth. Since both phenotypes show an additive effect on diauxic lag duration, they should not act serially. Since growth rate on cellobiose is unaffected by both mutation in the CelR EIIB domain and in spr0505, the most likely hypothesis is that both limit the functionality of the CelR regulator, possibly acting on the PRD domains, until a threshold is reached, beyond which the regulator is appropriately phosphorylated and becomes able to drive expression of the operon.

With reference to the FP376 mutant (glucose type PTS spr0505), the mathematical model identification results show that the smallest duration of diauxic lag produces the lowest value for 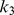 parameter for all initial concentrations. The analysis of the dynamic variables of the model result shows that the short diauxic lag is the result of two factors: the 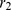 function high values, which means high bacterial growth rate, and high values for 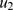 control enzyme induction, which means clearly that the spr0278-80-82 locus is transcribed promptly, resulting early in high level for enzyme PTS spr0278. Moreover similar values of 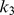 for all cellobiose initial concentration, indicate that the bacterial growth and transcription are not affected by cellobiose concentration in FP376.

In summary, our data demonstrate how mathematical modelling enabled us to resolve a biological phenomenon, indicating precisely which of the molecular changes is related to which phenotype. In particular, we report a conflicting regulatory mechanism, which in presence of high concentrations of cellobiose determines an extensive lag phase during diauxic growth. The regulatory conflict leading to delayed growth on cellobiose was found to depend on a second beta-glucoside PTS spr0505, which stimulated by its substrate and at high cellobiose concentrations delayed efficient activation of the CelR – PTS spr0278-80-82 mediated cellobiose uptake We are not yet able to resolve the molecular mechanism by which cellobiose utilisation is blocked. However we were able to show that genetic inactivation of the phosphorylable cysteine of the EIIB^GAT-like^ domain in the CelR regulator is involved in retardation of CelR functionality, irrespective of the action of spr0505. The biological reason for this conflicting regulatory mechanism is not clear, but could relate to rational task division between two transporters for similar substrates or to the control of a core genome PTS-spr0505 onto PTS-spr0278-80-82, being part of the accessory genome [5], [6].

## Materials and Methods

### Bacterial Strains, Media and Growth Condition

*S. pneumoniae* strains used in this work were the serotype 2 strain DP1004 (unencapsulated derivative of D39) [20], [21], the serotype 19F strain G54 and their isogenic mutants [22], [23]. The mutant for the beta-glucoside PTS EIIBCA^Glc^ spr0505 has been described [5]. Bacterial stock aliquots were obtained growing *S. pneumoniae* in tryptic soy broth (TSB; Liofilchem) at 37°C until the OD_590_ of 0.2, added of 20% glycerol and finally stored at −80°C. On solid media bacteria were grown in tryptic soy agar (TSA; Liofilchem) supplemented with 3% vol/vol horse blood. Growth curves were assayed in CAT medium composed of bacto casitone 10 g/l (Becton Dickinson), bacto yeast extract 1 g/l (Becton Dickinson), tryptone 5 g/l (Oxoid) and sodium chloride 5 g/l. Just before use, CAT medium was buffered adding 3% vol/vol of K_2_HPO_4_ 0.5 M [24]. Due to the presence of bacto yeast extract (Beckton Dickinson) the carbohydrate-unsupplemented CAT medium contained 0.16 g/l of total carbon source.

### Transfer of the Beta-glucoside Operon to G54

Marker-less transfer of the spr0274-0282 region into G54 strain was performed following standard protocols for pneumococcal transformation followed by selection on sugar substrates. Briefly, chromosomal DNA of the D39 derivative DP1004 and CSP1 (synthetic competence stimulating peptide 1; Inbios, Pozzuoli, Napoli, Italy) were added to competent cells in CAT medium supplemented with 1% of glucose [24]. After incubation at 37°C for 45 minutes in the presence of DNA, cells were diluted 1∶20 into liquid CAT medium supplemented with 1% of gentiobiose, fermented solely by the donor. For transformants selection, cultures were diluted after growth for 12 hours five times successively using 1∶100 dilutions into medium with the selecting sugar as sole carbon source. After five passages in selecting sugar the bacteria were plated on gentiobiose containing plates and colonies were checked by PCR for presence of the spr0274-0282 operon. One of the G54 derivatives containing the spr0274-0282 was named FP294.

### Mutant Construction

Site directed change of a single amino acid in the EIIB and EIIA domains of spr0279 was performed using a two-step transformation procedure with an *rpsL* cassette, named Janus [18]. Recombinant DNA constructs carrying two homology regions for chromosomal integration were obtained by gene SOEing [24]. The first step requested transformation into a streptomycin resistant strain of the Janus cassette carrying a wild type *rpsL* allele and a selectable kanamycin marker (*rpsL*, *aphIII*). This step resulted in disruption of the target gene on the chromosome. In the second transformation deletion of the cassette by homologous recombination restored streptomicin resistance and replaced the cassette with the recombinant gene with a single codon modification through a negative selection. For site directed mutagenesis two PCR fragments where joined utilising the central primers for the generation of the recombinant sequence containing the requested codon changes. The isogenic mutants created and the primers used for constructing the CelR EIIB_C414A_, EIIB_C414D_ and EIIA_H577A_ mutants of the spr0279 regulator are shown in Table 1 and Table S1. Clones obtained were controlled by sequencing and only clones without any additional polymorphism were selected for further work.

A double mutant for EIIBCA^Glc^ spr0505 and CelR EIIB_C414A_ was constructed by transforming a PCR with the PTS mutation [5] into competent cells of the CelR mutant. Construction of a mutant of the EIIA domain of spr0505 was constructed as above in a two step process taking advantage of the Janus cassette. The spr0505 gene is part of an operon with the downstream beta-glucosidase spr0506 and the EIIA domain of the transporter is encoded by the C-terminal part of the gene. The in frame deletion of the EIIA domain of spr0505 was constructed by deletion of a 297 bp internal fragment of spr0505 (the EIIA domain only) and fusing the part coding for the other two transporter domains to the terminal 115 bp of the gene in order to leave the intergenic region between spr0505 and spr0506 intact (Table S1).

### Growth on Beta-glucosides

*S. pneumoniae* strains were grown on TSA plates at 37°C in a CO_2_ enriched atmosphere for 18 hours. Bacteria were collected from agar plates with a swab and resuspended in CAT medium without supplemented carbohydrates at the OD_590_ of 0.2. The bacteria resuspension were diluted 1∶100 in CAT medium added of catalase 200 U/µl (Sigma; C9322) and a single carbohydrate as carbon source. For monitoring pneumococcal growth in different beta-glucosides, bacteria were grown in CAT medium supplemented with glucose (Panreac; 131341), gentiobiose (Carbosynth; OG05175) and cellobiose (Sigma; 22150). In order to compare the growth of wild type strain and spr0274-0282 operon complemented clone, DP1004 and FP294 were grown in different beta-glucosides, including amygdalin (Sigma; A6005). The wild type growth in presence of different concentration of beta-glucosides, was performed growing DP1004 in a serial two-fold dilutions ranging from 0,5% to 0,015% w/v. To evaluate the phenotypic behaviour of a single amino acid changes in beta-glucosides metabolism, the DP1004, its isogenic mutants in EIIB and EIIA domains of CelR regulator and PTS transporters, were grown in 1%, and 0.3% as final concentrations. The positive control was set up with the medium added of glucose, while the medium with no sugar supplemented was used as a negative control. Microtiter plates were sealed with gas permeable sealing membrane (Breath-Easy, BEM-1; Diversified Biotech, Boston, Ma) and incubated at 37°C for 18 hours or 24 hours in a thermostatic kinetic microplate reader (VERSAmax, Molecular Devices, Sunnyvale, Ca). Plates were shaken gently for 10 seconds prior to each reading; the absorbance at OD_590_ was measured every 10 minutes.

### Model Identification

The initial conditions of bacterial concentration 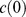, substrate concentrations 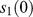, 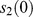 and enzyme concentrations 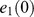, 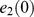 are specified by the experimental conditions. Numerical values for model parameters whose knowledge is well assessed, were taken from the literature [25]. This is the case for the yield coefficients of Monod 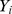, the Michaelis-Menten constants for enzymes 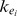, the basal rates of enzyme synthesis 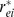, the first order enzyme decay constants 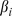 and the enzyme maximum rate constants 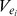. The parameter 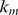 is taken from [9]. The remaining model parameters are stacked in a parameter vector denoted by 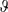. The only measured quantity is the bacterial concentration 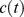 over a period of time corresponding to the growth phase. The unknown parameters have been estimated by minimizing weighted mean square error cost function:



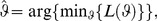
(9)


where:
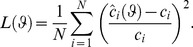
(10)In equation (10) 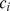 is the experimental measurement of bacterial concentration at time 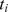, 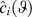 represents the bacterial concentration predicted by the model at the same time 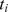, and *N* is the number of measurements collected in each experiment. The algorithm used for the cost function minimization is the Nelder-Mead algorithm developed in the optimization toolbox in Matlab R2010b, which is basically a nonlinear unconstrained optimization algorithm.

The sampling time for numerical simulations was fixed at 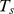 = 10 min according to the Kinetic Reader technical features (see subsection Growth on beta-glucoside). The value of *N* according to different growth period ranges from 57 to 90.

The numerical values of estimated parameters for the *wt* and mutants are shown in Tables 3 and 4 for a single concentration of cellobiose and gentiobiose. Parameter values for the *wt,* both on cellobiose and gentiobiose, are reported in Tables 5 and 6. Finally, estimated parameters for mutants FP411 FP376 and FP463 are reported in Tables 7, 8, and 9.

## Supporting Information

Table S1 Primers used for mutant construction and EIIA and EIIB sequencing.
(DOCX)
