# Supplementary material for: A Non-Linear Deterministic Model for Regulation of Diauxic Lag on Cellobiose by the Pneumococcal Multidomain Transcriptional Regulator CelR
Source: PLoS One. 2012 Oct 22;7(10):e47393. doi: 10.1371/journal.pone.0047393 (PMC3478281; doi:10.1371/journal.pone.0047393)
Supplement: Table S1 — Primers used for mutant construction and EIIA and EIIB sequencing. (DOCX) [file pone.0047393.s001.docx]

**SUPPORTING INFORMATION**

**Table S1:** Primers used for mutant construction and EIIA and EIIB sequencing.

| **Name** | **Sequence** | **Target region** | **Reference** |
| --- | --- | --- | --- |
| Kan5 | CCGTTTGATTTTTAATGGATAATG | *ami* promoter and *aphIII* | [1] |
| 7 | GGAAAGGGGCCCAGGTCTCT | Downstream of *rpsL* | [1] |
| LG041 | GAGCAAGTTCCAGTTCTTTTTG | EIIB domain | This study |
| LG042 | CATTATCCATTAAAAATCAAACGGAGTCTATTTCCTGAACTGG | EIIB domain - Janus cassette | This study |
| LG043 | GGAAAGGGGCCCAGGTCTCTGGTACAGAGGGAAAGGTTT | EIIB domain - Janus cassette | This study |
| LG044 | CTAACATTTCCTTTGCTACTTC | EIIB domain | This study |
| LG048 | AATCCCGTTGCGGCAATAGCAAGA | EIIB (Cys changed in Ala) | This study |
| LG049 | TCTTGCTATTGCCGCAACGGGATT | EIIB (Cys changed in Ala) | This study |
| LG060 | AATCCCGTTGCGTCAATAGCAAGA | EIIB (Cys changed in Asp) | This study |
| LG061 | TCTTGCTATTGACGCAACGGGATT | EIIB (Cys changed in Asp) | This study |
| LG054 | GTTTTGGATGAATGCCA | EIIA domain | This study |
| LG055 | CATTATCCATTAAAAATCAAACGGCATCATCTGATATTGACC | EIIA domain - Janus cassette | This study |
| LG056 | GGAAAGGGGCCCAGGTCTCTGAATACGCAAGTGGAGTG | EIIA domain - Janus cassette | This study |
| LG057 | GTCTAACAAACTCAGAACT | EIIA domain | This study |
| LG058 | GTTGAATAGGAGCCGGAACTGC | EIIA (His changed in Ala) | This study |
| LG059 | GCAGTTCCGGCTCCTATTCAAC | EIIA (His changed in Ala) | This study |
| LG062 | CTAAAGTAAGAGAAGTAACAAA | EIIB domain sequencing | This study |
| LG063 | GATGGAGATAGAAGAAATACT | EIIB domain sequencing | This study |
| LG064 | GCAAATATGGACAAGTTCT | EIIA domain sequencing | This study |
| LG065 | CCTTTGCTACTTCTAATAAT | EIIA domain sequencing | This study |
| AB040 | AATGTGGTGAACAGGTAGCAAT | spr0505 ko mutant | [2] |
| AB041 | ATCAAACGGATCCCCAGCTTG ACCAAGACTTTCAACATACCTC | spr0505 ko mutant | [2] |
| AB042 | CCTACGAGGAATTTGTATC ATTATCCCAAGTGAAGGTAAGG | spr0505 ko mutant | [2] |
| AB043 | ACCATCTTCATTATATGCTCCC | spr0505 ko mutant | [2] |
| AB126 | TCCAGCAATGTTAGGGTCAAA | spr0505 EIIA mutant | This study |
| AB132 | TCACATTATCCATTAAAAATCAAACGGT GGAATGATTTCTCCTGATATAG | spr0505 EIIA mutant | This study |
| AB128 | CGGGATCCCTTTCCTTATGCTTTT CTACGAAACAGTTACACCAGTA | spr0505 EIIA mutant | This study |
| AB129 | AGAGTTACAAGTGGTTCTATC | spr0505 EIIA mutant | This study |
| AB130 | GGAATGATTTCTCCTGATATAG | spr0505 EIIA mutant | This study |
|  |  |  |  |

Reference List

1. Sung CK, Li H, Claverys JP, Morrison DA (2001) An *rpsL* cassette, Janus, for gene replacement through negative selection in *Streptococcus pneumoniae*. Appl Environ Microbiol 67: 5190-5196.

2. Bidossi A, Mulas L, Decorosi F, Colomba L, Ricci S, et al., (2012) A functional genomics approach to establish the complement of carbohydrate transporters in *Streptococcus pneumoniae*. PLoS ONE 7: e33320.
